# Characterising ISWI chromatin remodeler in *Trypanosoma cruzi*

**DOI:** 10.1590/0074-02760190457

**Published:** 2020-05-18

**Authors:** Yirys Díaz-Olmos, Michel Batista, Adriana Ludwig, Fabricio K Marchini

**Affiliations:** 1 Fundação Oswaldo Cruz-Fiocruz, Instituto Carlos Chagas, Laboratório de Ciências e Tecnologias Aplicadas em Saúde, Curitiba, PR, Brasil; 2 Fundación Universidad del Norte, División Ciencias de la Salud, Barranquilla, Colombia; 3 Fundação Oswaldo Cruz-Fiocruz, Instituto Carlos Chagas, Plataforma de Espectrometria de Massas - RPT02H, Curitiba, PR, Brasil

**Keywords:** ISWI, chromatin remodeling, interacting partners, reverse genetics, p*Tc*GW plasmid vectors

## Abstract

**BACKGROUND:**

Imitation SWItch (ISWI) ATPase is the catalytic subunit in diverse chromatin remodeling complexes. These complexes modify histone-DNA interactions and therefore play a pivotal role in different DNA-dependent processes. In *Trypanosoma cruzi*, a protozoan that controls gene expression principally post-transcriptionally, the transcriptional regulation mechanisms mediated by chromatin remodeling are poorly understood.

**OBJECTIVE:**

To characterise the ISWI remodeler in *T. cruzi* (*Tc*ISWI).

**METHODS:**

A new version of p*Tc*GW vectors was constructed to express green fluorescent protein (GFP)-tagged *Tc*ISWI. CRISPR-Cas9 system was used to obtain parasites with inactivated *Tc*ISWI gene and we determined *Tc*ISWI partners by cryomilling-affinity purification-mass spectrometry (MS) assay as an approximation to start to unravel the function of this protein.

**FINDINGS:**

Our approach identified known ISWI partners [nucleoplasmin-like protein (NLP), regulator of chromosome condensation 1-like protein (RCCP) and phenylalanine/tyrosine-rich protein (FYRP)], previously characterised in *T. brucei*, and new components in *Tc*ISWI complex [DRBD2, DHH1 and proteins containing a domain characteristic of structural maintenance of chromosomes (SMC) proteins]. Data are available via ProteomeXchange with identifier PXD017869.

**MAIN CONCLUSIONS:**

In addition to its participation in transcriptional silencing, as it was reported in *T. brucei*, the data generated here provide a framework that suggests a role for *Tc*ISWI chromatin remodeler in different nuclear processes in *T. cruzi*, including mRNA nuclear export control and chromatin compaction. Further work is necessary to clarify the *Tc*ISWI functional diversity that arises from this protein interaction study.

*Trypanosoma cruzi* is a protozoan parasite causing Chagas disease, a neglected tropical disease from Latin America. In the last decades, this disease has become a public health problem in the developed world, because of globalisation and migration processes.^1^

In this pathogen, transcriptional regulation is a mechanism poorly understood. Because unusual characteristics of its genome, such as absence of classic RNA Polymerase II promoters and presence of polycistronic transcription of unrelated genes, the gene expression is thought to be regulated mainly at post-transcriptional level (RNA stability, translation, and protein stability) (reviewed in^2^). However, epigenetic control has also been involved as a mechanism of gene expression regulation in *T. cruzi* ^3^ ^,^ ^4^ with the presence of histone modifications and variant histones at the strand-switch regions.^5^ Besides, visible changes in the chromatin architecture occur during the life cycle of this parasite, which were associated with alterations in the transcription rate.^6^

In eukaryotes, chromatin remodeling is an essential mechanism to regulate DNA-dependent processes. This function requires the role of chromatin-modifying multiprotein complexes, which include ATP-dependent nucleosome-remodeling complexes and chromatin-modifying enzymes (*e.g.* histone acetyltransferase and histone deacetylase). The first category uses the energy of ATP hydrolysis to disrupt or alter the histone-DNA interaction, thus regulating many processes such as transcription, DNA repair, recombination and replication (reviewed in^7^). This motor activity is driven in all these remodeling complexes by an ATPase subunit that belongs to the SNF2 superfamily and comprises two regions, the SNF2_N and Helicase_C subdomains.^8^ Adjacent to these domains, additional domains are found that categorise each one of the remodeler families (SWI/SNF, ISWI, NURD/Mi-2/CHD and INO80 families).^7^

Imitation SWItch (ISWI)^9^ is one of the most diverse groups of chromatin remodelers. Nucleosome-remodeling complexes containing ISWI as the catalytic subunit have been identified in *Drosophila*, yeast, *Xenopus*, *Arabidopsis*, and mammals. In these organisms, several ISWI complexes are present, which are composed of distinct auxiliary subunits (two to five subunits) that specialise the remodeler for particular functions (reviewed in^10^). In *Trypanosoma brucei*, an early-branching eukaryote phylogenetically related to *T. cruzi*, ISWI remodeler (*Tb*ISWI) was identified and was suggested to be a transcriptional regulator at multiple Pol I-transcribed loci. *Tb*ISWI plays a role in the repression of Pol I-transcribed expression sites such as silent variant surface glycoprotein (VSG) genes, the procyclin loci and the rDNA transcriptional units.^11^ ^,^ ^12^ In *T. brucei*, ISWI associated partners were determined, and the three proteins found [nucleoplasmin-like protein (NLP), phenylalanine/tyrosine-rich protein (FYRP) and regulator of chromosome condensation 1-like protein (RCCP)] integrate the unique ISWI complex identified in this parasite. Knockdown of these proteins resulted in the derepression of genes in the Pol I-transcribed loci,^13^ the same phenotype observed in parasites with *Tb*ISWI depletion.

Unlike *T. brucei*, where the role of ATP-dependent chromatin remodeling complexes and its participation in the regulation of transcription started to be unraveled, there is limited information about the function of these complexes in *T. cruzi*. The main goal of this research was to characterise the ISWI protein in *T. cruzi* (*Tc*ISWI) using an updated set of plasmid vectors for *T. cruzi* (p*Tc*GW vectors, version 2.0) and different approaches to understand the function of this chromatin remodeler in this parasite. Our affinity purification-MS procedure allowed confirming the principal components of the ISWI complex in trypanosomatid parasites (NLP, RCCP and FYRP) and also identify new ISWI interacting proteins [DRBD2, DHH1 and proteins containing a domain characteristic of structural maintenance of chromosomes (SMC) proteins]. The identification of these new proteins, participating in the *Tc*ISWI complex, attributes novel functions to this chromatin remodeler and suggests that chromatin remodeling takes part in different processes in the nucleus of *T. cruzi*.

## MATERIALS AND METHODS

*TcISWI orthologous identification, protein domain analyses and sequence alignment* - To confirm that the *Tc*ISWI is a single copy gene, we performed blastn searches at TriTrypDB against Transcript database from distinct strains/assemblies of *T. cruzi*, using the nucleotide sequence of *Dm*28c clone (BCY84_16296) as a query.

Reciprocal Best Hit method was used to confirm the orthology of the *Tc*ISWI gene with known ISWI genes from other species. In this method, two proteins are considered orthologous if they can find each other as the best match when BLAST searches are performed. The protein sequence of *Tc*ISWI from *T. cruzi Dm*28c clone (TriTrypDB ID: BCY84_16296) was used as a query. Searches were carried out by BlastP tool in the NCBI database (https://blast.ncbi.nlm.nih.gov/Blast.cgi) against the model organisms *Homo sapiens*, *Xenopus laevis*, *Drosophila melanogaster*, *Arabidopsis thaliana* and *Saccharomyces cerevisiae*, and against the related species *T. brucei*. The reciprocal BlastP was performed in the TriTrypDB (https://tritrypdb.org/tritrypdb/) against *T. cruzi Dm*28c genome using as queries the sequences from the first hits obtained in the primary searches.

For sequence alignment, the *Tc*ISWI protein sequence was compared with the closest homologous sequences found in model organisms. The alignment was obtained using PSI-Coffee (in T-Coffee Server, http://tcoffee.crg.cat/), a tool to align distantly related proteins. Genedoc 2.7 was used for alignment visualisation and to determine the pairwise identity and similarity between the sequences.^14^

Conserved domains within the protein sequences of *Tc*ISWI and its partners were determined by CD-search tool using the Conserved Domains Database (CDD) at NCBI (https://www.ncbi.nlm.nih.gov/Structure/cdd/cdd.shtml) applying the default parameters. Phyre2 (http://www.sbg.bio.ic.ac.uk/phyre2/), a tool for remote homology detection and 3D structure prediction, was used to analyse the *Tc*ISWI protein sequence.

**Fig. 1:**
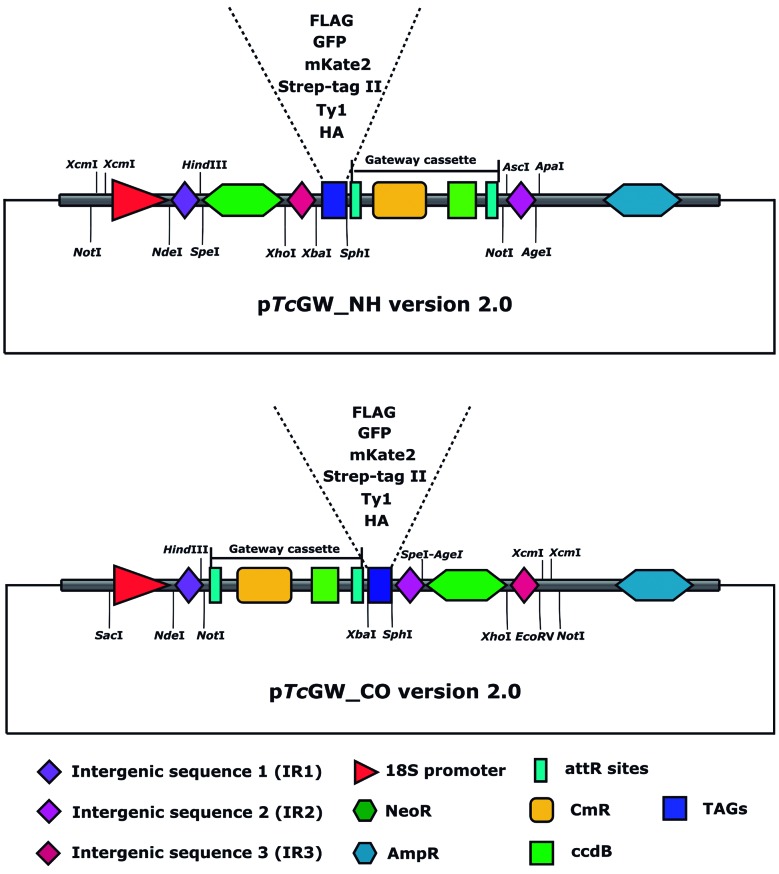
p*Tc*GW vectors version 2.0. Vector maps showing each regulatory element (promoter and intergenic sequences) to expression of proteins in *Trypanosoma cruzi* and other features such as resistance mark (NeoR), different options of tags, ampicillin resistance gene (AmpR) and Gateway cassette (for cloning the gene sequence encoding target protein) containing attR sites (recombination sites), chloramphenicol resistance mark (CmR) and ccdB gene. Above: vector for expression of tagged proteins at N-terminal; Below: vector for expression of tagged proteins at C-terminal end.

*pTcGW plasmid vectors version 2.0 for expression of tagged proteins in T. cruzi* - An upgraded version of the p*Tc*GW vectors^15^ ^,^ ^16^ was used in this study. To construct these plasmids, the cassettes for expressing proteins tagged at N-terminal or C-terminal end with FLAG tag were synthesised (GenScript) and cloned into pBlue Script II KS+. Then, these vectors were used as a template to replace the tag and the resistance marker (to hygromycin) using specific restriction sites, as shown in the vector maps (Fig. 1). Therefore, we generated twelve different vectors for expression of proteins in *T. cruzi* with fusion N- or C-terminal to the tags green fluorescent protein (GFP), mKate2, Strep-tag II, Ty1, and three tandem repetitions of HA or FLAG. The nucleotide sequences of the vectors were deposited at NCBI GenBank with accession numbers MT180298 (pTcHAN-CO), MT180299 (pTcHAN-NH), MT180300 (pTcFlagN-CO), MT180301 (pTcFlagN-NH), MT180302 (pTcStrepN-CO), MT180303 (pTcStrepN-NH), MT180304 (pTcGFPN-CO), MT180305 (pTcGFPN-NH), MT180306 (pTcmKateN-CO), MT180307 (pTcmKateN-NH), MT180308 (pTcTy1N-CO) and MT180309 (pTcTy1N-NH).

*Parasites and culturing* - *Dm*28c clone of *T. cruzi* was used in all experiments. Epimastigote forms were cultivated at 28ºC in Liver Infusion Tryptose (LIT) medium supplemented with 10% (v/v) foetal bovine serum (FBS), 100 μg mL^−1^ ampicillin and 100 μg mL^−1^ streptomycin. Epimastigote forms expressing GFP-tagged *Tc*ISWI protein were used to determine the subcellular localisation and to identify the partners of *Tc*ISWI. These parasites were maintained in the same medium previously specified supplemented with 250-500 μg mL^−1^ G418.

*Plasmid construction for expression of GFP-tagged TcISWI protein* - Epitope tagging of ISWI with GFP at the amino- (N-) or carboxy- (C-) terminus and episomal expression of the tagged protein was performed using the p*Tc*GW plasmids version 2.0 (p*Tc*GFPN-NH and p*Tc*GFPN-CO vectors) containing the GFP tag. First, the ISWI gene coding sequence (TriTrypDB ID: BCY84_16296) was ampliﬁed from *T. cruzi Dm*28c genomic DNA with primers carrying *att*B sites partial sequence [ISWI_F and ISWI_R primers; Supplementary data **(Table I)**]. To add the *att*B sites complete sequence, a second polymerase chain reaction (PCR) was performed with the primers *att*B_F and *att*Bw_R or *att*Bwo_R, which also adding or not stop codon for tagging to N- or C-terminal ends of the protein, respectively [Supplementary data **(Table I)**]. The cloning steps were carried out using Gateway® cloning system (Thermo Scientiﬁc). In brief, the PCR product was cloned into the pDONR221 vector and then transferred to the p*Tc*GFPN destination vector with N- or C-terminal fusion to GFP to obtain the p*Tc*GFPN-NH-ISWI (neomycin/G418 resistance, N-terminal tag) and p*Tc*GFPN-CO-ISWI (neomycin/G418 resistance, C-terminal tag) expression vectors. All reactions were performed as described in the manufacturers’ manuals. These plasmid vectors with the ISWI sequence cloned were confirmed by sequencing. To construct the vector expressing only GFP protein, we cloned the gene sequence encoding a hypothetical protein (TriTrypDB ID: BCY84_03386) in p*Tc*GFPN vector (N- and C-terminal fusion), and then we removed it using restriction digestion while preserving the *att*B recombination sites.

*Parasites transfection* - Nucleofection was used to transfect epimastigote forms as described by Pacheco et al.^17^ Briefly, 2 x 10^7^ epimastigotes in exponential growth phase were washed once with *Tb*BSF buffer (5 mM KCl, 0.15 mM CaCl_2_, 90 mM Na_2_HPO4, 50 mM HEPES, pH 7.3), centrifuged at 3,000 x g for 5 min to room temperature (RT) and then resuspended in the same buffer. Then, 10-20 µg of each plasmid DNA were added to the parasites and transfected using the Amaxa Nucleofector device, X-014 program. Transfected parasites were cultured in LIT medium and one-day post-transfection the medium was supplemented with 500 μg ml^−1^ G418. After four days, a 1:5 dilution was prepared and the culture was maintained during several weeks to selection. Three weeks post-transfection, G418-resistant parasites were obtained and thereafter these parasites were analysed by flow cytometry and western blot, as specified below, to confirm the expression of GFP-tagged *Tc*ISWI protein.

*Flow cytometry* - Fluorescent activated cell sorting (FACS) was used to enrich fluorescent populations in the cultures. Single cell sorting was used to obtain clonal populations of the parasite after implementing of CRISPR/Cas9 system to disrupt the *Tc*ISWI gene. For all cytometry assays, cells were previously washed once with PBS using centrifugation at 3,000 x g for 5 min and then resuspended in the same buffer. Fluorescence of the cultures was monitored using a FACS Canto II flow cytometer (BD Bioscience). Cell sorting used a FACSAria flow cytometer (Flow cytometry facility RPT08L / Carlos Chagas Institute ― Fiocruz, Paraná, Brazil). Data acquisition was carried out with the FACS Diva software and data analysis used FlowJo software v10 (https://www.flowjo.com/). In all the experiments, 20,000 events per analysis were collected.

*Immunoblotting* - Total protein extract (1 x 10^6^ parasites µL^-1^) was prepared from parasites expressing GFP-tagged *Tc*ISWI protein using sodium dodecyl sulfate-polyacrylamide gel electrophoresis (SDS-PAGE) sample buffer containing 40 mM Tris-HCl, 1% SDS, 2.5% β-mercaptoethanol, 6% glycerol, 0.005% bromophenol blue. After boiling at 95ºC for 10 min, 10 µL (1 x 10^7^ parasites) were loaded on 13% SDS-PAGE gel and the proteins were transferred to nitrocellulose membrane (GE Healthcare, PA, USA), blocked with 5% non-fat milk prepared in PBS/0.05% Tween 20, and incubated with rabbit polyclonal antibody (in-house product) against GFP (1:1000) for 1 h at RT. After three washing steps with PBS/0.05% Tween 20, the membrane was incubated with anti-rabbit alkaline phosphatase-conjugated IgG for 1 h at RT. Finally, after extensive wash steps, antigens were detected using a colorimetric assay (phosphatase assay).

*Indirect immunofluorescence assays* - *T. cruzi* epimastigotes with stable expression of GFP-tagged *Tc*ISWI were used to determinate the subcellular localisation of this protein. After fixation with 4% PFA for 30 min, 1 x 10^6^ parasites/well were washed with PBS and transferred to glass slides pre-treated with 0.1% poly-L-lysine. Parasites were permeabilised with a solution containing 0.5% Triton X-100 pH 8.0 for 5 min, washed once with PBS and incubated with blocking solution (3% BSA) for 1 h. Then, parasites were incubated for 1h at room temperature with a dilution 1:200 of mAbs GFP (Invitrogen - Thermo Fisher Scientific) prepared in 1.5% BSA, washed three times with blocking solution and incubated for 1 h at RT with anti-mouse IgG conjugated with Alexa Fluor 488 (Invitrogen - Thermo Fisher Scientific) diluted 1:600 in 1.5% BSA. After three washes with blocking buﬀer, followed by three washes with PBS and ﬁnally two washes in water, the slides were mounted with coverslips using Prolong Diamond (Invitrogen - Thermo Fisher Scientific) to visualise the nucleus and kinetoplast. Images were acquired on a Leica DMI6000B fluorescence inverted microscopy associated with Leica AF6000 deconvolution software (Microscopy Facility RPT07C / Carlos Chagas Institute - Fiocruz, Paraná, Brazil).

*In vitro metacyclogenesis* - *In vitro* differentiation of epimastigote forms to metacyclic parasites was performed as previously described.^18^ Briefly, epimastigotes in late exponential growth phase (5-8 x 10^7^ parasites mL^-1^) in LIT medium were harvested by centrifugation, washed once in PBS and once in triatomine artificial urine (TAU) medium (8 mM phosphate buﬀer pH 6.0, 190 mM NaCl, 17 mM KCl, 2 mM MgCl_2_, 2 mM CaCl_2_) and incubated in the same medium for 2 h at 28ºC at a density of 5 × 10^8^ cells ml^-1^. After this time, parasites were diluted 1:100 in TAU supplemented with 50 mM sodium glutamate, 10 mM L-proline, 2 mM sodium aspartate and 10 mM glucose (TAU3AAG), and maintained at 28ºC for 72 h. At this time, differential counting in a Neubauer hemocytometer was made to determinate the number of metacyclic forms in the culture supernatant. Experiments were performed in technical duplicate and biological triplicate, and the data were subjected to statistical t-test analysis, using the GraphPad PRISM© 7, Inc. software.

*Cell proliferation analyses* - Epimastigote cultures were established at an initial density of 1 x 10^6^ cells ml^-1^ and the population growth was monitored for eight days by cell counting using the automatised counter Z2 Particle Counter (Beckman Coulter). The settings were Threshold Low (TL) 2.6 µm, Threshold Up (TU) 5.2 µm, considering the particles above TL. The cell proliferation analyses were performed in technical triplicate, and the data were subjected to statistical t-test analysis, using the GraphPad PRISM© 7, Inc. software.

*TcISWI gene editing by CRISPR/Cas9* - The *Tc*ISWI gene was edited using the CRISPR/Cas9 approach, as previously reported by Medeiros et al.^19^ to produce parasites with the inactive *Tc*ISWI gene. The editing system used *Staphylococcus aureus* Cas9 (SaCas9) enzyme, delivery of ribonucleoprotein complex and repair of DNA double-strand breaks by homology-directed repair. DNA repair template added stop codons (in the three different frames) and the sequence for M13_R primer annealing to the editing *Tc*ISWI gene in the parasite genome.

Eukaryotic Pathogen CRISPR guide RNA/DNA Design Tool (http://grna.ctegd.uga.edu/) was used to identify the single-guide RNA (sgRNA) target sequence and to design DNA repair template sequence [Supplementary data **(Table I)**]. sgRNA preparation and SaCas9/sgRNA ribonucleoprotein complex assembly were performed according to Medeiros et al.^19^ Briefly, sgRNA preparation was made by *in vitro* transcription (IVT) using as template a DNA containing scaffold sequence specific for SaCas9, sgRNA sequence and T7 promoter sequence [Supplementary data **(Table I)** for details of the primer sequences used to amplify the DNA template]. Before the assembly of the ribonucleoprotein complex, sgRNA was incubated at 90ºC for 5 min and slowly cooling to RT for 15 min. Equimolar amounts of SaCas9 and sgRNA (1:1 ratio) were incubated at room temperature for 15 min previous to transfect the complex in 1 x 10^6^ epimastigotes by nucleofection as previously specified, this time using the program U-033 on Amaxa Nucleofector device. Before transfection, 20 µg repair template was added. Transfected parasites were cultured in 25-cm^2^ cell culture flasks in 5 mL of complete LIT medium. Ribonucleoprotein complex nucleofection was carried out two times in intervals of one or two days. After, the parasites were let for two or three days before performing single cell sorting. Gene editing was confirmed by PCR directly from liquid culture using the WT_F and M13_R primers [Supplementary data **(Table I)**].

*Cell lysis for affinity purification assay* - Cells were lysed by cryogenic grinding. Briefly, frozen cell pellets obtained from exponential growth epimastigote culture were achieved by dropping a cell slurry into liquid nitrogen. Cell lysis was performed by cryogenic disruption, frozen cells were ground into a powder using a Retsch MM400 device with 5 mL stainless steel milling cup. Cell breakage cycles were run to 30 Hz for 2 min each and repeated five times. Before use and between each grinding cycle, milling cup, balls and spatulas were cooled in liquid nitrogen to keep the cells frozen. The resulting cell powder was stored at -80ºC.

*Affinity isolation of protein complex* - Affinity medium consisted of nanobodies from llama specific against GFP (clone LaG-16-G_4_S-LaG-2) coupled to Dynabeads M-270 epoxy (Invitrogen). Expression and purification of nanobodies used the protocol described by Fridy et al.^20^ The coupling followed the procedure specified by Obado et al.^21^ using 30 mg beads and 300 µg nanobodies in each coupling assay. Nanobodies-coupled beads were stored in PBS, 50% glycerol at -20ºC.

For the affinity isolation of *Tc*ISWI and its partners, it was evaluated different extraction buffer conditions as recommended by La Cava et al.^22^ In each affinity capture test, 600 μL extraction buffer was added to 100 mg cell powder. After, one step of low power microprobe sonication was applied to disperse and homogenise aggregates. After homogenisation and extraction, clarification of the extract was performed by centrifugation at 20,000 x g for 10 min at 4ºC. Affinity media (5 µL nanobodies-coupled Dynabeads), pre-washed three times with 1 mL extraction buffer, were added to the clarified cell extracts and incubated at 4ºC for 30 min with rotary mixing. Then, the beads were collected using a magnet and washed three times with the same extraction buffer. During the second wash, the beads were transferred to a fresh tube. This step is needed to minimise the presence of contaminants, nonspecifically adsorbed to the plastic surfaces, in the final eluted. Protein complexes were eluted with 40 mM Tris-HCl pH 8.0, 2% SDS at 72ºC for 20 min. After that, the final eluted was collected, incubated at 95ºC for 5 min in SDS-PAGE sample buffer, analysed on 13% SDS-PAGE gels and visualised by silver staining before protein digestion for liquid chromatography-mass spectrometry (LC-MS)/MS analysis. Affinity purification assay was performed in triplicate using as bait GFP-tagged *Tc*ISWI in the NH and CO extremity, and for each assay frozen powder of parasites expressing only the GFP tag was used as control.

*LC-MS/MS analysis* - After separation of the proteins eluted from the affinity purification procedure by SDS-PAGE, each gel lane was split in three fractions, excised out of gel and each fraction was cut in 1 x 1 mm pieces. After destaining, proteins were reduced with 0.01 M DTT and alkylated with 0.05 M iodoacetamide before protein digestion using 12.5 ng mL^-1^ trypsin (Promega, V5113) diluted in 50 mM ammonium bicarbonate (ABC) at 37ºC for 18 h. Then, peptides were extracted from gel matrix, concentrated by vacuum centrifugation and desalted using C18 columns (Stagetip). Peptides were analysed by LC-MS/MS using an Easy-nLC 1000 (Thermo Scientific) coupled online to an LTQ Orbitrap XL ETD (Thermo Scientific) (Mass Spectrometry Facility RPT02H / Carlos Chagas Institute - Fiocruz, Paraná, Brazil). Peptide samples were fractionated via reverse-phase chromatography using a 15 cm fused silica capillary containing 3 µm C18 particles (ReproSil-Pur C18-AQ, Dr Maisch GmbH). The chromatography was carried out at 250 nL min^-1^ with a gradient of 5 to 40% of MeCN in 0.1% formic acid, 5% DMSO for 60 min. Mass spectrometer was set to data-dependent acquisition mode, operating to alter automatically between MS1 and MS2 acquisition. MS1 spectra (*m/z* 300-2,000) were acquired in the Orbitrap analyser with a resolution of 60,000. The top 10 most intense precursor ions were sequentially isolated, fragmented by CID and detected in the LTQ (linear trap quadrupole). Protein validation, quantification and identification used the MaxQuant platform (version 1.6.3.4) set to default parameters. Proteins were identified by an automatic search against a *T. cruzi* protein databank (TriTrypDB-41_TcruzicruziDm28c_AnnotatedProteins) containing 20,257 sequences. Contaminant proteins (human keratins, BSA and porcine trypsin) and the reverse of all the sequences, including contaminants, were also included in the search and manually removed from the list of identifications. Protein quantification was based on the unique peptide identified and on the protein intensity, obtained by the sum of the respective MS1 peptide 3D peak area. The mass spectrometry proteomics data have been deposited to the ProteomeXchange Consortium via the PRIDE^23^ partner repository with the dataset identifier PXD017869.

## RESULTS

*General features of pTcGW vectors version 2.0* - Here we evaluated a new version of the p*Tc*GW vectors which use the Gateway Technology. In this new version (Fig. 1), we maintained the promoter region for RNA polymerase I (rDNA 18S) and replaced the intergenic sequences which are localised adjacent to the resistance gene and the cloning site (Gateway cassette) in the vectors. The selection of these new intergenic sequences [Supplementary data **(Table II)**] was based on proteomic and transcriptomic data (unpublished data) and considered the constitutive expression of genes in epimastigote and metacyclic trypomastigote stages of *T. cruzi*. Thus, these intergenic regions could enable the use of these vectors in different life cycle stages of *T. cruzi*; however, we did not address this possibility here. p*Tc*GW version 2.0 expands the tag options available [Fig. 1, Supplementary data **(Table III)**], so increasing their applicability in different methods such as co-expression and co-localisation assays using fluorescent proteins (GFP and mKate2). Another new useful characteristic of these vectors is the flexibility to clone 3’ and 5’ UTR regions through TA cloning (using *Xcm*I sites) and Gateway recombination. This feature allows the construction of knockout cassettes for gene deletion by homologous recombination. Finally, *Not* I restriction sites (Fig. 1) were added to the vectors to allow linearising the plasmids before transfection, when cassettes for gene knockout are used.

**Fig. 2:**
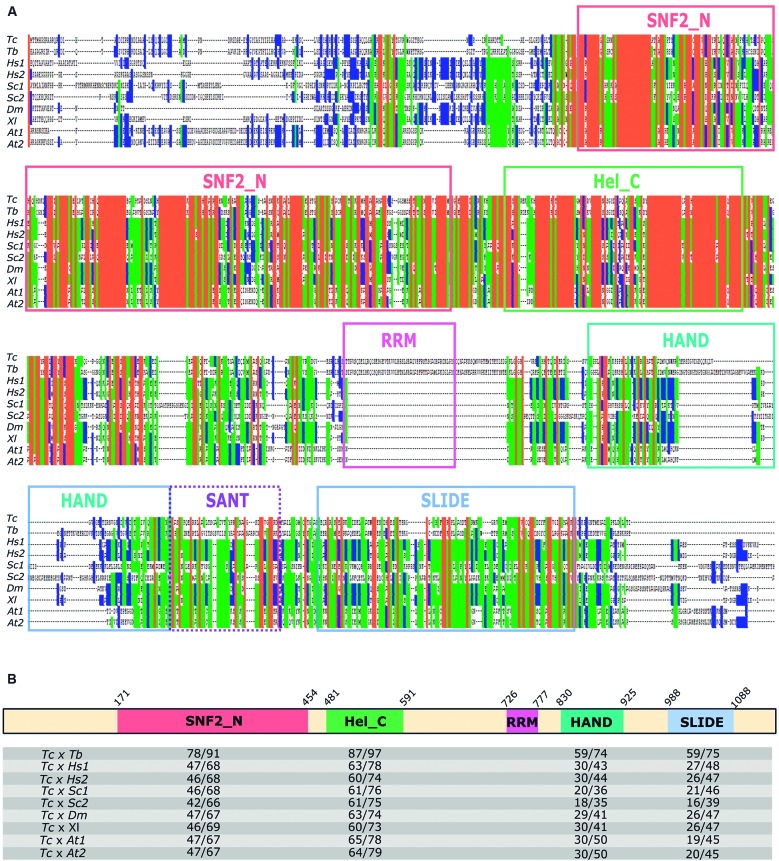
comparison of Imitation SWItch (ISWI) orthologs sequences and *Tc*ISWI domain architecture analysis. (A) Multiple sequence alignment of complete ISWI proteins as obtained by PSI-coffee and visualised by Genedoc 2.7. Colours on the sequences denote as follows: orange, 100% conserved residues; green, > 60% conserved residues; blue, > 40% conserved residues. Protein domains are highlighted with boxes. The domain SANT, represented as a dashed outline was not predicted with CD-search in *Trypanosoma cruzi*. The sequences displayed in the alignments are as follows: *Tc*: *T. cruzi* (BCY84_16296); *Tb*: *T. brucei* (Tb927.2.1810); *Hs*1: *Homo sapiens* SMARCA1 protein (NP_001269804.1); *Hs*2: *H. sapiens* SMARCA5 protein (NP_003592.3); *Sc*1: *Saccharomyces cerevisiae* Isw2p protein (NP_014948.1); *Sc*2: *S. cerevisiae* Isw1p protein (NP_009804.1); *Dm*: *Drosophila melanogaster* (NP_523719.1); *Xl*: *Xenopus laevis* (XP_018097820.1); *At*1: *Arabidopsis thaliana* protein 11 (NP_001189826.1) and *At*2: *A. thaliana* protein 17 (NP_568365.2). (B) Domain architecture analysis of *Tc*ISWI protein. The predicted domains are shown in coloured boxes with the indication (above) of start and end amino acid (aa) residues position of each domain. Below are shown the pairwise aa identity/similarity values (expressed as a percentage) between *Tc*ISWI protein and the orthologous for each domain. For the HAND domain, the low conserved region in the alignment (corresponding the aa 873-889 of *Tc*ISWI) was removed for identity/similarity determination.

*TcISWI: an ISWI family chromatin remodeler in T. cruzi* - In eukaryotes, ISWI ATPases are highly conserved proteins. *T. cruzi* encodes an ATPase of SNF2 superfamily, orthologous of ISWI proteins in other eukaryotes [Supplementary data **(Table IV)**]. This protein with 1113 amino acids (aa) presents highly similar regions when compared with its orthologues, as evidenced after multiple protein sequence alignment [Fig. 2A; see Supplementary data **(Fig. 1)** for sequence alignment details]. Interestingly, sequence alignment analysis revealed the presence of a 75-aa region (position 726-801 in *Tc*ISWI) exclusive to ISWI of trypanosomatids (*T. cruzi* and *T. brucei*). Analysis of this region resulted in the identification of an RNA recognition motif (RRM) (position 726-777), a prevalent RNA-binding fold among proteins with a role in RNA metabolism processes.^24^

The ISWI family ATPases are distinguished by the presence of an ATPase subunit that belongs to the SNF2 superfamily. This family contains an ATPase domain that comprises two subdomains, SNF2_N and Helicase_C. In the C-terminal half of the protein, the HAND, SANT (SWI3, ADA2, N-CoR and TFIIIB) and SLIDE (SANT-like ISWI domain) domains are found, which allow the interaction of this ATPase with DNA and histones.^25^ Domain architecture analysis of *Tc*ISWI identified all these domains, except the SANT domain (Fig. 2B). However, the secondary structure prediction showed a high similarity in this region with c2y9zA template, an ISWI protein (ISW1a) from *Saccharomyces cerevisiae* [Supplementary data **(Fig. 2)**].

**Fig. 3:**
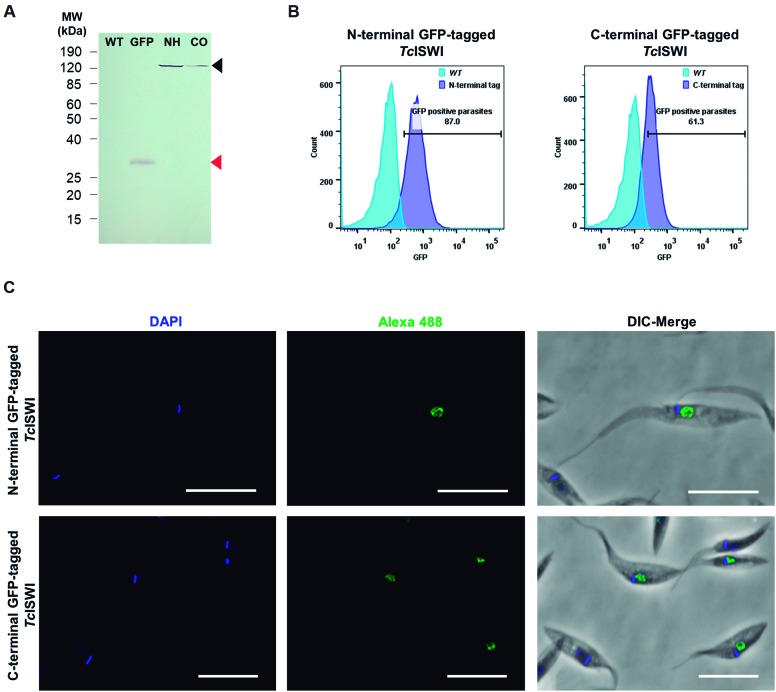
expression and subcellular localisation of GFP-tagged *Tc*ISWI protein in epimastigotes. (A) Western blot using total protein extract of wild type (WT) parasites, parasites expressing green fluorescent protein (GFP) and parasites expressing *Tc*ISWI with N-terminal (NH) or C-terminal (CO) fusion to GFP. (B) Flow cytometry of G418-resistant parasites. Histograms showing the percentage of GFP positive parasites (histogram dark blue) compared with WT parasites (histogram clear blue). (C) Subcellular localisation of GFP-tagged *Tc*ISWI in epimastigote forms of *Trypanosoma cruzi* evaluated by indirect immunofluorescence microscopy. GFP-tagged *Tc*ISWI parasites were treated with mAbs GFP and then with a secondary antibody coupled to AlexaFluor-488 (green). Parasites were labeled with DAPI for DNA staining. Merged images with differential interference contrast (DIC) are presented in the last column. Bar: 10 µm.

*Expression and subcellular localisation of GFP-tagged TcISWI protein* - Before determining the subcellular localisation of ISWI protein in epimastigote forms of *T. cruzi*, we confirmed the expression of the protein with N- and C-terminal fusion to GFP by flow cytometry and western blot. These results would allow us also to validate the functionality of p*Tc*GW vectors version 2.0 for tagged protein expression in *T. cruzi*. As shown in Fig. 3A, western blot assay revealed the presence of a 156 kDa band as expected for GFP-tagged *Tc*ISWI. Besides, flow cytometry analysis confirmed fluorescence associated with the population of G418-resistant parasites (Fig. 3B). Once the expression of the GFP-tagged *Tc*ISWI was confirmed, we assessed its subcellular localisation by indirect fluorescence microscopy. ISWI is an ATPase participating in chromatin remodeling complexes, and, as we expected, the tagged protein (both C- and N-terminal GFP fusion) localised in the nucleus of epimastigote forms of *T. cruzi* (Fig. 3C). This result allowed us to not only know the localisation of ISWI remodeler in *T. cruzi* but also confirm the functionality of p*Tc*GW vectors version 2.0 for expression of tagged proteins in this parasite. In addition, this result indicated that the presence of the tag did not affect the adequate localisation of *Tc*ISWI allowing additional studies on the role of *Tc*ISWI in these transfected cells.

*GFP-tagged TcISWI expressing and TcISWI editing cells evaluation* - We intended to obtain knockout parasites of the *Tc*ISWI gene using CRISPR-Cas9 tool to evaluate the participation of this protein in different cellular processes. We first confirmed that this gene is presented as a single copy in the *T. cruzi Dm*28c genome [Supplementary data **(Table V)**]. After several attempts to edit the two alleles of this gene, only parasites with one edited allele were achieved as assessed via PCR [Supplementary data **(Fig. 3A)**]. In order to verify the influence of both GFP-tagged *Tc*ISWI and the heterozygous knockout on the cell proliferation rate, parasite cultures were followed for eight days and no difference in growth rate was observed compared with wild type parasites [Supplementary data **(Fig. 3B, C)**]. We also evaluated possible alterations in the metacyclogenesis process in these parasites considering *Tc*ISWI protein presented regulated phosphorylation sites during this differentiation stage.^26^ We did not observe significant changes in the number of metacyclic trypomastigote cells obtained after *in vitro* metacyclogenesis [Supplementary data **(Fig. 3D)**].

*ISWI partners in T. cruzi* - In eukaryotes, ISWI ATPase exists as part of protein complexes with a vast functional diversity, which is determined by the associated proteins. To characterise ISWI binding proteins, we used parasites expressing the chimera protein GFP-tagged *Tc*ISWI. We applied an optimised affinity purification workflow including cryogenic lysis, evaluation of different lysis buffer, isolation of tagged complex using nanobody-conjugated magnetic beads and finally protein identification by LC-MS/MS. Each one of these steps offers distinct advantages to other approaches^22^ that finally allow efficient isolation and accurate characterisation of protein complexes while minimising the number of nonspecific associations.

We chose the tagged version of *Tc*ISWI protein as bait in our affinity purification approach instead of the endogenous protein or fusion protein expressed from the endogenous locus. This choice was based on: (i) the relative easiness to obtain parasites expressing the recombinant protein fused to the reporter from episomal vectors; (ii) the difficulty for genetic manipulation in *T. cruzi* by homologous recombination and (iii) the availability of a nanobody with high specificity and affinity against GFP to ensure efficient and clean purification. In addition, the use of the GFP tag allows obtaining information regarding both protein localisation and interactions without depending on the production of specific antibodies against the target protein.

**Fig. 4:**
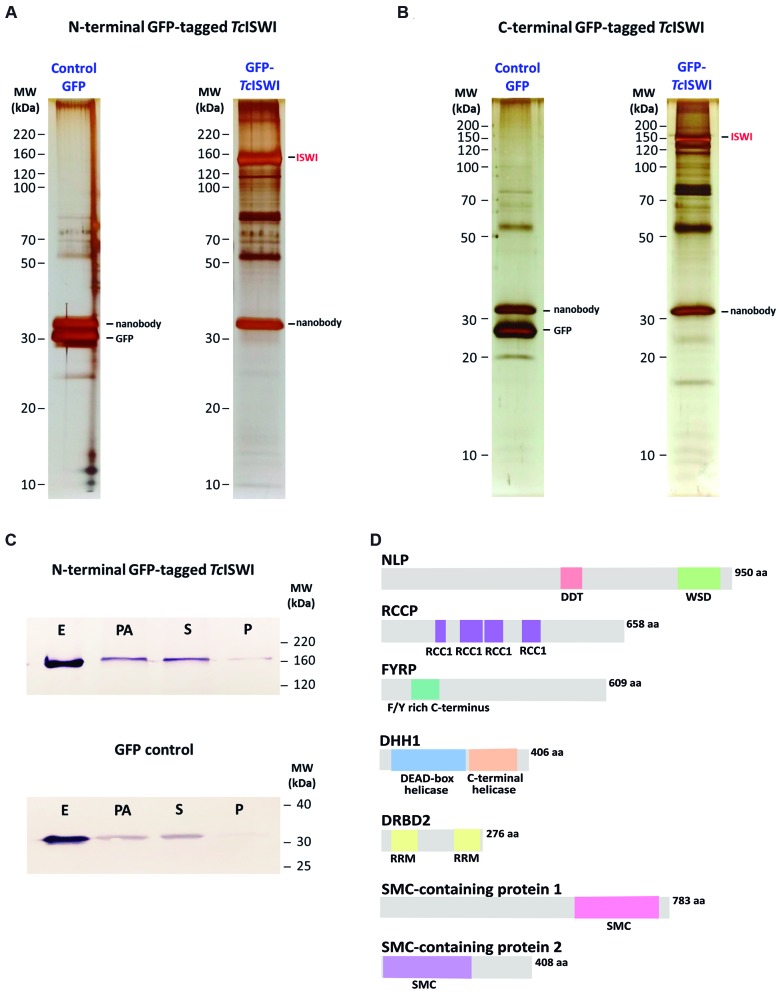
affinity purification procedure for identification of *Tc*ISWI partners. Analysis by sodium dodecyl sulfate-polyacrylamide gel electrophoresis (SDS-PAGE) gels stained with silver of extracts obtained after affinity purification procedure using the N-terminal green fluorescent protein (GFP)-tagged *Tc*ISWI (A) and the C-terminal GFP-tagged *Tc*ISWI (B) as bait. Control samples gels (Control GFP) correspond to eluted proteins using GFP as bait. The bands of GFP-tagged *Tc*ISWI, GFP and nanobody are indicated. Size markers are indicated on the left of each gel. (C) Western blot showing the enrichment of N-terminal GFP-tagged *Tc*ISWI (above) and GFP (below) during the affinity purification procedure. Samples were collected of clarified cell lysate (P: pellet; S: supernatant), supernatant post-incubation with nanobody coupling beads (PA) and final elute (E). Size markers are indicated on the right. (D) Domain architecture analysis of *Tc*ISWI partners. The predicted domains are shown in colored boxes with the description under each box. The TriTrypDB ID of proteins are available at Table.

After evaluated five types of lysis buffers [Supplementary data **(Fig. 4)**], we chose the optimised lysis buffer containing 40 mM Hepes-Na pH 7.4, 50 mM citrate, 1 mM MgCl_2_, 10 µM CaCl_2_, 0,1% Triton X-100, 10% glycerol and proteases inhibitor in the affinity purification procedures. This buffer allowed to obtain prominent bands with difference between the control and the target samples. As there is no previous data on the possible interference of GFP tag, both NH and CO-fusion, to the *Tc*ISWI complex stability, we evaluated the ISWI partners using both versions of the tagged protein. Consequently, we could diminish the chance of no identification of partners due to the presence of GFP tag, which might affect the protein-protein interactions. Three independent affinity purification assays were performed using each version of GFP-tagged *Tc*ISWI and in each assay frozen powder of parasites expressing only GFP was used as control. As shown in Fig. 4A-B, a range of bands of co-isolated proteins was visualised in the GFP-tagged *Tc*ISWI samples and GFP controls. A prominent band was observed at 150 kDa in the target samples, corresponding to the GFP-tagged *Tc*ISWI protein (Fig. 4C). Also, immunoblotting assay was used to evaluate the enrichment of the protein during the affinity purification (Fig. 4C). For that, we collected samples during the procedure to evaluated the presence of *Tc*ISWI protein preferentially in the supernatant (lane S, Fig. 4C) after clarification of the cell lysed and its enrichment in the final eluted (lane E, Fig. 4C). Although a portion of *Tc*ISWI did not bind to the beads (lane PA, Fig. 4C), the affinity purification procedure via GFP tag resulted in an efficient isolation of GFP-tagged *Tc*ISWI and GFP control. Then we followed with the identification of *Tc*ISWI interacting partners by LC-MS/MS. The raw data obtained in the LC-MS/MS is available in: https://drive.google.com/open?id=1jjpa7pYNIlwvdnbkG8swrurfmRfNA8MX.

To analyse the data generated by LC-MS/MS, initially in the MaxQuant output, we filtered the noise (unspecific interactions) considering absence or presence of the identified proteins in the controls and the target samples, respectively. We considered prey proteins that fitting the criteria of 0 unique peptides in the controls and with more than one unique peptide in the target samples (Table). We also considered quantitative information from LC-MS/MS analysis (intensity) and we calculated the intensity fold change in target samples relative to the controls. An arbitrary intensity, between “0” and the minor intensity value obtained, were attributed to non-identified proteins in the controls (Table). Through this data analysis flow, we identified three known ISWI binding partners previously defined as the single major ISWI complex in *T. brucei*.^13^ These proteins are NLP, RCCP and FYRP. Domain analysis using the CDD database evidenced the presence of conserved domains in these proteins (Fig. 4D).

**TABLE:** Proteins co-isolated with green fluorescent protein (GFP)-tagged *Tc*ISWI and identified by mass spectrometry

Gene ID	Protein description	CO Ctrl_1	CO Ctrl_2	CO Ctrl_3	NH Ctrl_1	NH Ctrl_2	CO Test_1	CO Test_2	CO Test_3	NH Test_1	NH Test_2	NH Test_3	Log10 (controls mean intensity)	Log10 (tests mean intensity)	Intensity fold-change
BCY84_16296	transcription activator (TcISWI)*	0	0	0	0	0	58	40	49	43	53	48	2	9.7	7.7
BCY84_11771	hypothetical protein (RCCP)**	0	0	0	0	0	12	9	13	13	13	10	2	8.6	6.6
BCY84_16205	unspecified product (NLP)**	0	0	0	0	0	17	15	19	15	18	15	2	8.3	6.3
BCY84_13416	hypothetical protein (FYRP)**	0	0	0	0	0	11	14	20	14	15	13	2	7.7	5.7
BCY84_13929	RNA-binding protein (DRBD2)***	0	0	0	0	0	6	1	5	5	8	10	2	7.1	5.1
BCY84_18524	hypothetical protein (SMC domain containing protein 1)***	0	0	0	0	0	1	4	2	2	12	10	2	6.8	4.8
BCY84_14589	hypothetical protein (SMC domain containing protein 2)***	0	0	0	0	0	2	1	1	2	3	3	2	6.3	4.3
BCY84_19601	unspecified product (DHH1)***	0	0	0	0	0	3	2	4	2	2	1	2	6.3	4.3

Note: table presents the preys identified with 0 unique peptide in the controls (Ctrl) and more than 1 unique peptide in the target samples (Test) for both NH and CO versions of GFP-tagged *Tc*ISWI. The average intensity Log10 for each prey protein and the fold-change relative to the controls are shown. An arbitrarily intensity, between “0” and the minor intensity value obtained, were attributed to non-identified proteins in the controls. *: bait protein; **: Imitation SWItch (ISWI) partners previously identified in *Trypanosoma brucei*; ***: new partners identified in this study. In total five control samples and six target samples using the bait protein (three replicates for both, NH and CO versions of GFP-tagged *Tc*ISWI) were analysed. Protein description was based on information available in the TriTrypDB or obtained after domain analysis or literature review.

In our partner assay, NLP, RCCP and FYRP were identified with the highest number of unique peptides, corresponding to the top values in the list of identified partners (Table). This data indicates these proteins are strong partners in the *Tc*ISWI complex. NLP, RCCP and FYRP are proteins exclusive of kinetoplastids (as previously referenced;^13^ and confirmed by using BlastP searchers) while containing domains characteristic to other eukaryotic proteins that bind chromatin. Among these proteins, only NLP presents domains frequently found in the accessory subunits of the ISWI-like remodelers in other eukaryotes. These domains are DDT (DNA binding homeobox and Different Transcription factors) and WSD (Williams-Beuren syndrome DDT motif) domains (Fig. 4D). DDT domain is found in different transcription and chromosome remodeling factors.^27^ A conserved biochemical function of the SLIDE domain of ISWIs in eukaryotes is its binding to DDT‐domain containing proteins.^28^

*T. cruzi* RCCP, like its orthologue in *T. brucei*, presents four RCC1 repeats (Fig. 4D). RCC1 repeats are present in the RCC1 superfamily, being repetitive sequences of 51-68 aa residues each. At the functional level, RCC1 is the best-characterised member of the family. RCC1 is a guanine nucleotide exchange factor (GEF) for Ran (*Ra*s-related *n*uclear). Guanosine triphosphatase (GTPase) Ran controls nucleocytoplasmic transport, mitotic spindle formation, and nuclear envelope assembly. These functions rely on the association of RCC1 with chromatin.^29^ On the other hand, FYRP is a protein presenting an “FY-rich” domain, which is a poorly characterised phenylalanine/tyrosine-rich region of around 50 aa residues that are found in a variety of chromatin-associated proteins.^30^

In addition to these proteins, our partner assay also identified new potential *Tc*ISWI interacting proteins. Conserved domains were also identified in these proteins as specified in Fig. 4D. DRBD2 protein is the orthologue of Gbp2 protein in yeast, a protein involved in mRNA quality control and export.^31^ DHH1 is an RNA helicase that in eukaryotes, including trypanosomes, has been involved in multiple RNA metabolism-related processes and accumulates in stress granules, processing bodies and nuclear periphery granules (NPGs).^32^ ^,^ ^33^

The two remaining *Tc*ISWI partners are proteins containing a domain characteristic of SMC proteins. Members of this protein family are implicated in several activities that modulate chromosome structure and participate in the segregation and condensation of these structures from bacteria to humans (review in^34^). These potential *Tc*ISWI partners, however, have no clear homologs in non-kinetoplastid species (as verified by BlastP searches against the NCBI nr database - data not shown). The gene BCY84_18524 (hypothetical protein) is found only in trypanosomatids, and its protein sequence is weakly conserved among species. The presence of this gene is indicated only in the species from the *Trypanosoma* genus and in *Blechomonas ayalai* in the TriTrypDB BCY84_18524 gene information. BlastP searches revealed hits with low significant (e-value ~ 10^-4^) in the other trypanosomatid species; however, we can confirm the orthology by a reciprocal blast and by confirming they are in synteny with BCY84_18524 [TriTrypDB IDs of ortholog genes are available in Supplementary data **(Table VI)**]. The gene BCY84_14589 (hypothetical protein) is found in all trypanosomatids and *Bodo saltans* (a free-living kinetoplastid). We refer to these proteins as SMC domain containing protein 1 and SMC domain containing protein 2, respectively.

Four additional proteins (TriTrypDB IDs: BCY84_18615, BCY84_02420, BCY84_02988 and BCY84_05332) also fulfilled the filtering criteria (Raw data is available from: https://drive.google.com/open?id=1jjpa7pYNIlwvdnbkG8swrurfmRfNA8MX). However, these proteins were not considered potential partners of *Tc*ISWI because they are mostly referenced as non-nuclear proteins and there is no information supporting their association with ISWI protein.

## DISCUSSION

Chromatin is a dynamic structure that in response to environmental, metabolic and development changes suffers conformational alterations, which regulate the gene expression. One of the mechanisms to alter chromatin structure and to allow its dynamism are the ATP-dependent chromatin remodeling processes.^7^ ISWI ATPase is the catalytic subunit acting in protein complexes that use ATP hydrolysis to change histone-DNA interactions through disrupting, assembling or displacement nucleosomes.^7^ In this way, these complexes regulate DNA-dependent processes as transcription (activation or repression), replication, recombination, repair, among others.^35^ ^,^ ^36^ ^,^ ^37^ In this study, we investigated the role of ISWI protein (*Tc*ISWI) in the protozoan *T. cruzi*, whose function remains unexplored.

Here, we also validated the functionality of p*Tc*GW vectors version 2.0 for the expression of tagged proteins in *T. cruzi*. In addition to offering a platform to the determination of subcellular localisation, co-localisation assays and expression of fusion proteins in *T. cruzi*, this new upgrade, like previous versions, ensures a fast and efficient cloning system with the advantage of allowing easy construction of cassettes for gene knockout by homologous recombination. Besides, the old intergenic regions, present in p*Tc*GW versions 1.0 and 1.1,^15^ ^,^ ^16^ were replaced by three new ones aiming to increase the possibility of tagged proteins expression in all life stages of *T. cruzi*. Nevertheless, this was not addressed here. We verified these vectors through successful expression and subcellular localisation of *Tc*ISWI and confirmed its usefulness to the isolation of tagged proteins and their interacting complexes by affinity purification-MS assay.

In *T. cruzi*, a parasite that controls the gene expression primarily at post-transcriptional level, there is very little direct evidence of the participation of chromatin remodeling mediated by ATP-dependent complexes as a mechanism of cellular processes regulation. So far, only one protein has been suggested as a chromatin dynamic remodeler in *T. cruzi*, possibly participating in a chromatin remodeling complex. This protein, BDF2 (bromodomain factor 2), presents a bromodomain that by phylogenetic analysis clustered with SWI/SNF family-related bromodomains of chromatin remodeling complexes. *Tc*BDF2 interacts with acetylated histones and accumulated in parasites treated with UV irradiation suggesting a role in chromatin dynamic.^38^ Histone modifying enzymes also participate in the dynamic regulation of chromatin and have been the object of several studies in *T. cruzi*.^39^ ^,^ ^40^

To unravel the role of *Tc*ISWI in chromatin dynamics in *T. cruzi*, one of the adopted approaches was the generation and evaluation of an ISWI knockout parasite by CRISPR-Cas9. However, after several attempts, the edition of the second allele was not achieved. This result suggests an essential role of this protein in epimastigote forms of *T. cruzi.* This was also proposed for the orthologous protein in *T. brucei* ^11^ and other eukaryotic organisms.^41^ Knockdown of ISWI protein (*Tb*ISWI) in *T. brucei* led to a diminished growth of the parasite,^11^ while for *T. cruzi*, we observed that the presence of only one active allele in the parasite was apparently enough to carry out the cellular function of this gene since the parasite growth was not altered. Also, there was no modification on the *in vitro* metacyclogenesis process. The essentiality of the *Tc*ISWI gene and the limited toolbox to make conditional knockdown in *T. cruzi* limited our attempts to determine the participation of *Tc*ISWI in different key cellular processes.

In an effort to provide functional information of *Tc*ISWI in a parasite where the transcriptional regulation mechanism mediated by chromatin remodeling is poorly understood, we used affinity purification-MS approach to determine the *Tc*ISWI interacting proteins. Our assay identified the previously reported partners from *T. brucei*, NLP, RCCP and FYRP^13^ and potential new *Tc*ISWI partners. The identification of the same ISWI partners in these two species corroborates the participation of these proteins as components of ISWI complex in trypanosomatids. These ISWI binding proteins seem to be specific components of the ISWI complex in Kinetoplastida, an evolutionary lineage very divergent from the other eukaryote organisms. Due to the conservation of this complex, we can suggest *Tc*ISWI could also have a role in the maintenance of a repressive state of the chromatin structure in silent regions of the genome transcribed by Pol I as it was previously described for *Tb*ISWI. However, in *T. cruzi*, genes involved in pathogenicity (trans-sialidases and mucins, among others) are transcribed by Pol II. Interestingly, *Tb*ISWI was found enriched at transcriptional strand switch regions (SSRs), sites of Pol II transcription initiation and termination, as determined by ChIP analysis.^12^ ^,^ ^13^ Likewise, it was observed a tendency of all members of *Tb*ISWI complex to localise in these regions.

In higher eukaryotic organisms, ISWI ATPase forms several complexes, each one with functions defined by the associated subunits. In *T. brucei*, *Tb*ISWI forms a single predominant *Tb*ISWI complex. Stanne et al.^13^ suggested that this simplicity of the *Tb*ISWI complex is caused by the relative lack of transcriptional control in this parasite. However, the authors did not rule out the presence of relatively minor subcomplexes. In fact, our partners assay identified new potential ISWI partners in *T. cruzi*. This finding suggests that besides its role in transcriptional repression, as it was determined in *T. brucei*, it is possible to consider new functions to *Tc*ISWI. The identification of additional partners in our assay could be explained by the differences in the stringency of the techniques used. To identify the ISWI partners in *T. brucei*, the authors used tandem affinity purification, a method that uses multiple isolation steps via different tags. This procedure is useful for achieving cleaner purifications, allowing the isolation of strong interactors with fewer non-specific interactions, however impacting the preservation of weak protein-protein interactions. In our assay, we used a complex isolation workflow including cryomilling and optimisation of the extraction buffer. The combination of these methods allows for preserving labile interactions.

Among the new ISWI partners, we identified DRBD2 and DHH1 proteins. Both proteins have been involved in processes associated with mRNA metabolism in *T. brucei* and *T. cruzi* ^31^ ^,^ ^42^ and are present in NPGs (nuclear periphery granules) in *T. brucei*.^33^ ^,^ ^43^ These granules function as an mRNA nuclear export control system, avoiding unprocessed mRNAs to reach translation^33^. Strikingly, *Tc*ISWI orthologue in yeast (ISW1) was described as an mRNP nuclear export surveillance factor that retains export-incompetent transcripts near their transcription site.^44^ An interesting point to highlight here is the finding of an RRM domain in *Tc*ISWI. RRM fold is a domain that interacts with RNA and proteins and is present in proteins involved in RNA metabolism.^24^ Corroborating this data, these new potential *Tc*ISWI partners were also identified in a proteomic analysis of chromatin-associated proteins in *T. cruzi*,^45^ despite reports showing a cytoplasmic localisation for them.

Our partners assay also identified two hypothetical proteins (BCY84_18524 and BCY84_14589) containing domains characteristic of SMC proteins which are implicated in several processes that modulate higher-order chromatin structure from bacteria to humans (review in^34^). Interestingly, in *Drosophila*, ISWI regulates higher-order chromatin structure^36^ and the interaction of SMC proteins with ISWI ATPase was reported in humans.^46^ These findings provide valuable insights on the possibility of the *Tc*ISWI complex participate in the changes of the chromatin architecture observed during the life cycle of *T. cruzi*.^6^

Altogether, our findings and the hypotheses raised here based on the literature sustain a model whereby *Tc*ISWI could be forming different complexes and operates in different nuclear processes in *T. cruzi*, including repression of transcription, mRNA biogenesis surveillance mechanism and chromatin compaction. The data presented here can drive further studies to better characterise *Tc*ISWI, precisely determining its function in this early-branching eukaryote.

## References

[1] Castillo-Riquelme M (2017). Chagas disease in non-endemic countries. Lancet Glob Health.

[2] Clayton C (2019). Regulation of gene expression in trypanosomatids living with polycistronic transcription. Open Biol.

[3] Alsford S, du Bois K, Horn D, Field MC (2012). Epigenetic mechanisms, nuclear architecture and the control of gene expression in trypanosomes. Expert Rev Mol Med.

[4] Martinez-Calvillo S, Romero-Meza G, Vizuet-de-Rueda JC, Florencio-Martinez LE, Manning-Cela R, Nepomuceno-Mejia T (2018). Epigenetic regulation of transcription in trypanosomatid protozoa. Curr Genomics.

[5] Respuela P, Ferella M, Rada-Iglesias A, Åslund L (2008). Histone acetylation and methylation at sites initiating divergent polycistronic transcription in Trypanosoma cruzi. J Biol Chem.

[6] Elias MC, Marques-Porto R, Freymüller E, Schenkman S (2001). Transcription rate modulation through the Trypanosoma cruzi life cycle occurs in parallel with changes in nuclear organisation. Mol Biochem Parasitol.

[7] Clapier CR, Cairns BR (2009). The biology of chromatin remodeling complexes. Annu Rev Biochem.

[8] Tyagi M, Imam N, Verma K, Patel AK (2016). Chromatin remodelers: We are the drivers!!. Nucleus.

[9] Ito T, Bulger M, Pazin MJ, Kobayashi R, Kadonaga JT (1997). ACF, an ISWI-containing and ATP-utilizing chromatin assembly and remodeling factor. Cell.

[10] Dirscherl SS, Krebs JE (2004). Functional diversity of ISWI complexes. Biochem Cell Biol.

[11] Hughes K, Wand M, Foulston L, Young R, Harley K, Terry S (2007). A novel ISWI is involved in VSG expression site downregulation in African trypanosomes. EMBO J.

[12] Stanne TM, Kushwaha M, Wand M, Taylor JE, Rudenko G (2011). TbISWI regulates multiple polymerase I (Pol I)-transcribed loci and is present at Pol II transcription boundaries in Trypanosoma brucei. Eukaryot Cell.

[13] Stanne TM, Narayanan MS, Ridewood S, Ling A, Witmer K, Kushwaha M (2015). Identification of the ISWI chromatin remodeling complex of the early branching eukaryote Trypanosoma brucei. J Biol Chem.

[14] Nicholas KB, Nicholas HB (1997). GeneDoc: a tool for editing and annotating multiple sequence alignments. Embnet News.

[15] Batista M, Marchini FK, Celedon PA, Fragoso SP, Probst CM, Preti H (2010). A high-throughput cloning system for reverse genetics in Trypanosoma cruzi. BMC Microbiol.

[16] Kugeratski FG, Batista M, Inoue AH, Ramos BD, Krieger MA, Marchini FK pTcGW plasmid vectors 1 (2015). 1 version a versatile tool for Trypanosoma cruzi gene characterisation. Mem Inst Oswaldo Cruz.

[17] Pacheco-Lugo L, Díaz-Olmos Y, Sáenz-García J, Probst CM, DaRocha WD (2017). Effective gene delivery to Trypanosoma cruzi epimastigotes through nucleofection. Parasitol Int.

[18] Bonaldo MC (1988). Cell-substrate adhesion during Trypanosoma cruzi differentiation. J Cell Biol.

[19] Medeiros LCS, South L, Peng D, Bustamante JM, Wang W, Bunkofske M (2017). Rapid, selection-free, high-efficiency genome editing in protozoan parasites using. CRISPR-Cas9 ribonucleoproteins. mBio.

[20] Fridy PC, Li Y, Keegan S, Thompson MK, Nudelman I, Scheid JF (2014). A robust pipeline for rapid production of versatile nanobody repertoires. Nat Methods.

[21] Obado SO, Field MC, Chait BT, Rout MP. (2016). High-efficiency isolation of nuclear envelope protein complexes from trypanosomes. In: Shackleton S, Collas P, Schirmer EC, editors. The nuclear envelope.

[22] LaCava J, Molloy KR, Taylor MS, Domanski M, Chait BT, Rout MP (2015). Affinity proteomics to study endogenous protein complexes pointers, pitfalls, preferences and perspectives. BioTechniques.

[23] Perez-Riverol Y, Csordas A, Bai J, Bernal-Llinares M, Hewapathirana S, Kundu DJ (2019). The PRIDE database and related tools and resources in 2019: improving support for quantification data. Nucleic Acids Res.

[24] Maris C, Dominguez C, Allain FH (2005). The RNA recognition motif, a plastic RNA-binding platform to regulate post-transcriptional gene expression. FEBS J.

[25] Grüne T, Brzeski J, Eberharter A, Clapier CR, Corona DF, Becker PB (2003). Crystal structure and functional analysis of a nucleosome recognition module of the remodeling factor ISWI. Mol Cell.

[26] Amorim JC, Batista M, da Cunha ES, Lucena ACR, Lima CVP, Sousa K (2017). Quantitative proteome and phosphoproteome analyses highlight the adherent population during Trypanosoma cruzi metacyclogenesis. Sci Rep.

[27] Doerks T, Copley R, Bork P (2001). DDT a novel domain in different transcription and chromosome remodeling factors. Trends Biochem Sci.

[28] Aravind L, Iyer LM (2012). The HARE-HTH and associated domains novel modules in the coordination of epigenetic DNA and protein modifications. Cell Cycle.

[29] Hadjebi O, Casas-Terradellas E, Garcia-Gonzalo FR, Rosa JL (2008). The RCC1 superfamily from genes, to function, to disease. Biochim Biophys Acta.

[30] Balciunas D, Ronne H (2000). Evidence of domain swapping within the Jumonji family of transcription factors. Trends Biochem Sci.

[31] Wippel HH, Malgarin JS, Inoue AH, Leprevost FDV, Carvalho PC, Goldenberg S (2019). Unveiling the partners of the DRBD2-mRNP complex, an RBP in Trypanosoma cruzi and ortholog to the yeast SR-protein Gbp2. BMC Microbiol.

[32] Holetz FB, Alves LR, Probst CM, Dallagiovanna B, Marchini FK, Manque P (2010). Protein and mRNA content of TcDHH1-containing mRNPs in Trypanosoma cruzi DHH1-mRNPs in Trypanosoma cruzi. FEBS J.

[33] Goos C, Dejung M, Wehman AM, M-Natus E, Schmidt J, Sunter J (2019). Trypanosomes can initiate nuclear export co-transcriptionally. Nucleic Acids Res.

[34] Hirano T (2005). SMC proteins and chromosome mechanics from bacteria to humans. Philos Trans R Soc Lond B Biol Sci.

[35] Aydin ÖZ, Vermeulen W, Lans H (2014). ISWI chromatin remodeling complexes in the DNA damage response. Cell Cycle.

[36] Corona DFV, Siriaco G, Armstrong JA, Snarskaya N, McClymont SA, Scott MP (2007). ISWI regulates higher-order chromatin structure and histone H1 assembly in vivo. PLoS Biol.

[37] Poot RA, Bozhenok L, van den Berg DLC.Steffensen S.Ferreira F.Grimaldi M (2004). The Williams syndrome transcription factor interacts with PCNA to target chromatin remodelling by ISWI to replication foci. Nat Cell Biol.

[38] Villanova GV, Nardelli SC, Cribb P, Magdaleno A, Silber AM, Motta MC (2009). Trypanosoma cruzi bromodomain factor 2 (BDF2) binds to acetylated histones and is accumulated after UV irradiation. Int J Parasitol.

[39] Campo VA (2017). Comparative effects of histone deacetylases inhibitors and resveratrol on Trypanosoma cruzi replication, differentiation, infectivity and gene expression Int J Parasitol. Drugs Drug Resist.

[40] Ramos TCP, Nunes VS, Nardelli SC, Pascoalino BS, Moretti NS, Rocha AA (2015). Expression of non-acetylatable lysines 10 and 14 of histone H4 impairs transcription and replication in Trypanosoma cruzi. Mol Biochem Parasitol.

[41] Deuring R, Fanti L, Armstrong JA, Sarte M, Papoulas O, Prestel M (2000). The ISWI chromatin-remodeling protein is required for gene expression and the maintenance of higher order chromatin structure in vivo. Mol Cell.

[42] Kramer S, Queiroz R, Ellis L, Hoheisel JD, Clayton C, Carrington M (2010). The RNA helicase DHH1 is central to the correct expression of many developmentally regulated mRNAs in trypanosomes. J Cell Sci.

[43] Kramer S, Marnef A, Standart N, Carrington M (2012). Inhibition of mRNA maturation in trypanosomes causes the formation of novel foci at the nuclear periphery containing cytoplasmic regulators of mRNA fate. J Cell Sci.

[44] Babour A, Shen Q, Dos-Santos J, Murray S, Gay A, Challal D (2016). The chromatin remodeler ISW1 is a quality control factor that surveys nuclear mRNP biogenesis. Cell.

[45] de Jesus TCL, Calderano SG, Vitorino FN, Llanos RP, Lopes MC, de Araújo CB (2017). Quantitative proteomic analysis of replicative and nonreplicative forms reveals important insights into chromatin biology of Trypanosoma cruzi. Mol Cell Proteomics.

[46] Hakimi MA, Bochar DA, Schmiesing JA, Dong Y, Barak OG, Speicher DW (2002). A chromatin remodeling complex that loads cohesin onto human chromosomes. Nature.

