# Supplementary material for: Characterising ISWI chromatin remodeler in Trypanosoma cruzi
Source: Mem Inst Oswaldo Cruz. 2020 May 18;115:e190457. doi: 10.1590/0074-02760190457 (PMC7233268; doi:10.1590/0074-02760190457)
Supplement: Supplementary file 1 [file 1678-8060-mioc-115-e190457-s.pdf]

TABLE I

DNA oligonucleotides used for polymerase chain reactions (PCRs) and CRISPR-Cas9 based genome edition

Oligo name	Sequence (5′-3′)	Description
ISWI_F	**GGCTCCACC**ATGATGACGACTATGCATCC	Primers used to amplify the *Tc*ISWI gene with the *att*B sites partial sequence (in bold)
ISWI_R	**TGGGTGGATYCA**ATCGGTCGTAAGTGAATCC	
attB_F	**GGGGACAAGTTTGTACAAAAAAGCA**GGCTCCACCATG	Primers used to add both the remaining sequence of *att*B sites and the sequence encoding or not the stop codon (in bold and in italic, respectively)
attBw_R	**GGGGACCACTTTGTACAAGAAAGC**TGGGTGGAT*TCA*	
attBwo_R	**GGGGACCACTTTGTACAAGAAAGC**TGGGTGGAT*CCA*	
sgRNA_ISWI_F	GAGAATTG**TAATACGACTCACTATAGGGAGA**G*ACTGAGGCCTTATCAAATTGA*GTTTTAGTACTCTG-TAATTTTAGGTATGAGGTAGACGAAAATTGTA	sgRNA sequence (T7 promotor in bold, target sequence in the *Tc*ISWI gene in italic and scaffold partial sequence is underlined)
Donor_ISWI	AAAGAGACCCAGCAACCAGTTCACCCCCTC**caggaaacagctatgac***ctatctatcta*AATTTGATAAGGCCTCA-GTTTGCCTCGTAT	Donor sequence (homology arms are underlined, M13_R primer sequence in bold and in tandem stop sequences in italic)
SaCas9scaf_R	AAAAAAATCTCGCCAACAAGTTGACGAGAT	Reverse primer used together with sgRNA_ISWI_F to amplify the sgRNA sequence
Scaffold template	GTTTTAGTACTCTGTAATTTTAGGTATGAGGTAGACGAAAATTGTACTTATACCTAAAATTACAGAA-TCTACTAAAACAAGGCAAAATGCCGTGTTTATCTCGTCAACTTGTTGGCGAGATTTTTTT	DNA template used to amplify the sgRNA_ISWI complete sequence that after was used for IVT (*in vitro* transcription)
WT_F	ATGATGACGACTATGCATCC	Primers used to amplify wild type genotype
WT_R	CAGTTCACCCCCTCAATTTG	
M13R-pUC(-40)	CAGGAAACAGCTATGAC	Primer used together with WT_F to confirm ISWI gene editing

TABLE II

Description of regulatory elements of p*Tc*GW vectors version 2.0 for expression of tagged proteins in *Trypanosoma cruzi*

Regulatory elements	Description	Size (bp)
18S promoter	Pol I promoter sequence of 18S rDNA	617
Intergenic sequence 1 (IR1)	BCY84_19573 gene downstream intergenic sequence	505
Intergenic sequence 2 (IR2)	BCY84_11588 gene downstream intergenic sequence	421
Intergenic sequence 3 (IR3)	BCY84_11584 gene downstream intergenic sequence	318

CC BY

TABLE III

Description of the p*Tc*GW 2.0 fusion tags

Tags	Description
GFP	Fluorescent protein (714 bp, 238 aa)
mKate2	Fluorescent protein (696 bp, 232 aa)
Strep-tag II	Synthetic peptide, WSHPQFEK
Ty1	10-residue peptide epitope, EVHTNQDPLD
HA	Peptide epitope with 3 in tandem repetition, YPYDVPDYAG-YPYDVPDYAGS-YPYDVPDYA
FLAG	Peptide epitope with 3 in tandem repetition, DYKDHDG-DYKDHDI-DYKDDDDK

TABLE IV

*Tc*ISWI orthologous identification

Organism	Query cover	E - value	Identity (%)	Description	ID (NCBI)	Best reciprocal hit in TriTrypDB	E-value
Trypanosoma brucei TREU927	98	0.0	65.45	transcription activator putative	XP_951514.1	BCY84_16296	0.0
Arabidopsis thaliana	73	0.0	49.46	chromatin-remodeling protein 11	NP_001189826.1	BCY84_16296	0.0
Arabidopsis thaliana	74	0.0	49.46	chromatin remodeling factor 17	NP_568365.2	BCY84_16296	0.0
Drosophila melanogaster	66	0.0	50	imitation SWI, isoform A	NP_523719.1	BCY84_16296	0.0
Xenopus laevis	75	0.0	48.66	PREDICTED: SWI/SNF-related matrix-associated actin-dependent regulator of chromatin subfamily A member 5	XP_018097820.1	BCY84_16296	0.0
Homo sapiens	67	0.0	49.82	probable global transcription activator SNF2L1 isoform c (SMARCA1/SNF2L)	NP_001269804.1	BCY84_16296	0.0
Homo sapiens	65	0.0	48.48	SWI/SNF-related matrix-associated actin-dependent regulator of chromatin subfamily A member 5 (SMARCA5/SNF2H)	NP_003592.3	BCY84_16296	0.0
Saccharomyces cerevisiae	60	7e-176	40.45	DNA translocase (Isw2p)	NP_014948.1	BCY84_16296	7e-175
Saccharomyces cerevisiae	46	2e-172	49.33	chromatin-remodeling ATPase ISW1 (Isw1p)	NP_009804.1	BCY84_16296	4e-180

Note: Table shows the best hits obtained in the specified organism genomes when the *Tc*ISWI protein sequence was used in BlastP searches. The reference proteins database (refseq_ proteins) was used in the searches against model organisms. As expected by the literature, two ISWI paralogs were found in *Homo sapiens*, *Saccharomyces cerevisiae* and *Arabidopsis thaliana*. The best hit in reciprocal BlastP searches against *Trypanosoma cruzi* genome (*Dm*28c clone 2017) in TriTrypDB was the *Tc*ISWI (BCY84_16296). Additional significant hits in each species were found; however, they represent other SNF2 superfamily members rather than ISWI proteins.

TABLE V

Confirmation of *Tc*ISWI as a single copy gene in different strains of *Trypanosoma cruzi*

Organism	E - value	Description	ID (TriTrypDB)	BlastP Best reciprocal hit in *H. sapiens**	Description	E-value
*Trypanosoma_cruzi_Dm*28c_2017	0.0	transcription activator putative	BCY84_16296	NP_001269804.1	probable global transcription activator SNF2L1	0.0
	6e-07	helicase	BCY84_11667	NP_006653.2	helicase SRCAP	1e-111
	4e-3	DNA excision/repair protein SNF2	BCY84_12024	NP_060139.2	DNA excision repair protein ERCC-6-like	1e-98
*Trypanosoma_cruzi_Dm*28c_2018	0.0	transcription activator	C4B63_13g40	NP_001269804.1	probable global transcription activator SNF2L1	0.0
	5e-07	DNA excision repair protein (fragment)	C4B63_40g120	NP_006653.2	helicase SRCAP	1e-112
	3e-3	DNA excision/repair protein SNF2	C4B63_69g25	NP_060139.2	DNA excision repair protein ERCC-6-like	1e-98
Trypanosoma cruzi Brazil A4	0.0	chromatin-remodeling complex atpase chain iswi	TcBrA4_0005130	NP_001269804.1	probable global transcription activator SNF2L1	0.0
	5e-07	SWI/SNF-related helicase, putative	TcBrA4_0025880	NP_006653.2	helicase SRCAP	5e-112
	3e-3	DNA excision/repair protein SNF2, putative	TcBrA4_0095670	NP_060139.2	DNA excision repair protein ERCC-6-like	2e-98
Trypanosoma cruzi CL Brener Esmeraldo-like	0.0	transcription activator, putative	TcCLB.510257.139	XP_016885240.1	probable global transcription activator SNF2L1	4e-08
Trypanosoma cruzi CL Brener Non-Esmeraldo-like	0.0	chromatin-remodeling complex atpase chain iswi ISWI	TcCLB.509213.170	NP_001269804.1	probable global transcription activator SNF2L1	0.0
	8e-09	SWI/SNF-related helicase, putative	TcCLB.506155.70	NP_006653.2	helicase SRCAP	3e-111
	2e-3	DNA excision/repair protein SNF2, putative	TcCLB.504797.70	NP_060139.2	DNA excision repair protein ERCC-6-like	3e-99

Note: Table shows the best hits (with e-value < 10^{-3}) obtained in blastn at TriTrypDB against Transcript database from distinct strains/assemblies of *T. cruzi*, using the nucleotide sequence of the *Tc*ISWI gene (BCY84_16296) as a query. NCBI BlastP best hit against *H. sapiens* RefSeq-protein database is also shown. As can be seen in the results, *Tc*ISWI single copy is confirmed in all assemblies. The second hit in each assembly corresponds to another chromatin remodeler functional group since the best match in *H. sapiens* corresponds to the SRCAP gene belonging to the SWR1 group.

TABLE VI

Orthologs of *Trypanosoma cruzi* BCY84_18524 gene identified after BlastP searches and synteny confirmation

TriTrypDB ID	Organism
CFAC1_170020200	*Crithidia fasciculata strain Cf-Cl*
EMOLV88_190014600	*Endotrypanum monterogeii strain LV88*
LAEL147_000285200	*Leishmania aethiopica L147*
LAMA_000299600	*Leishmania amazonensis MHOM/BR/71973/M2269*
LBRM2903_190013800	*Leishmania braziliensis MHOM/BR/75/M2903*
LENLEM3045_190015200	*Leishmania enriettii strain LEM3045*
LGELEM452_190014700	*Leishmania gerbilli strain LEM452*
LINF_190015900	*Leishmania infantum JPCM5*
LMARLEM2494_190015300	*Leishmania sp. MAR LEM2494*
LMJLV39_190016200	*Leishmania major strain LV39c5*
LMJSD75_190016100	*Leishmania major strain SD 75.1*
LPAL13_000018500	*Leishmania panamensis MHOM/COL/81/L13*
LPAL13_000018600	*Leishmania panamensis MHOM/COL/81/L13*
LPMP_190920	*Leishmania panamensis strain MHOM/PA/94/PSC-1*
LTRL590_190014700	*Leishmania tropica L590*
LTULEM423_190014600	*Leishmania turanica strain LEM423*
LbrM.19.1340	*Leishmania braziliensis MHOM/BR/75/M2904*
LbrM.19.2.001340	*Leishmania braziliensis MHOM/BR/75/M2904 2019*
LdBPK.19.2.001030	*Leishmania donovani strain LV9*
LdBPK_191030.1	*Leishmania donovani BPK282A1*
LdCL_190015600	*Leishmania donovani CL-SL*
LmjF.19.1060	*Leishmania major strain Friedlin*
LmxM.19.1060	*Leishmania mexicana MHOM/GT/2001/U1103*
LpyrH10_04_1830	*Leptomonas pyrrhocoris H10*
Lsey_0030_0060	*Leptomonas seymouri ATCC 30220*
LtaP19.0970	*Leishmania tarentolae Parrot-TarII*

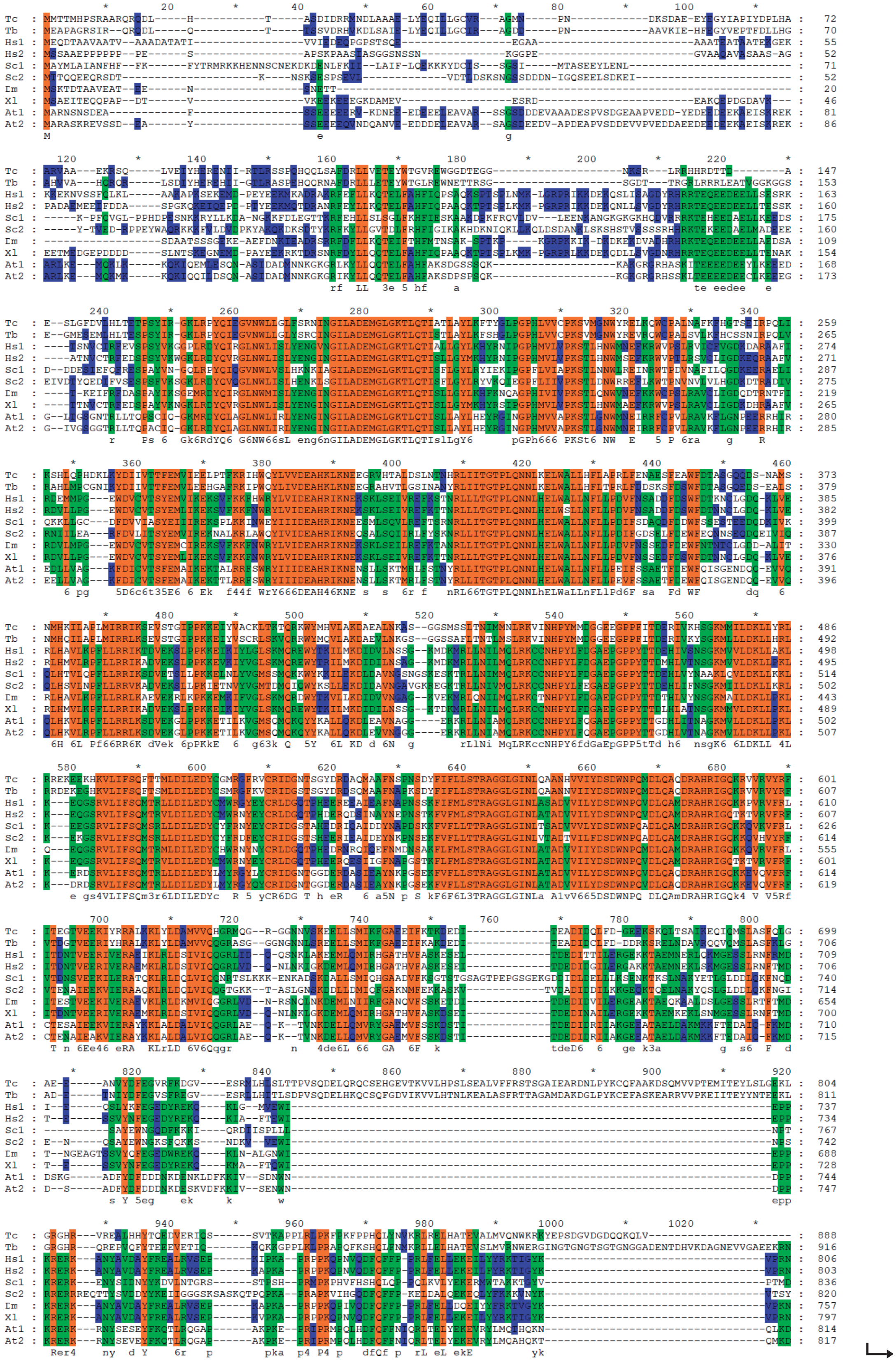

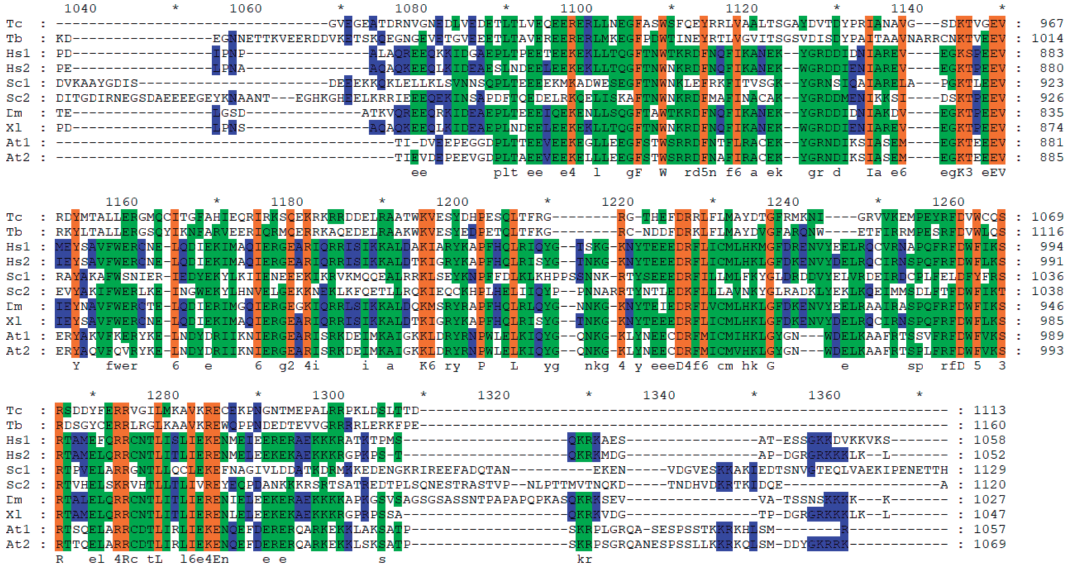

Fig. 1: multiple sequence alignment of complete Imitation SWItch (ISWI) proteins as obtained by PSI-coffee and visualised by Genedoc 2.7. Colours on the sequences denote as follows: orange, 100% conserved residues; green, > 60% conserved residues; blue, > 40% conserved residues. The sequences displayed in the alignments are as follows: *Tc*: *Trypanosoma cruzi* (BCY84_16296); *Tb*: *Trypanosoma brucei* (Tb927.2.1810); *Hs1*: *Homo sapiens* SMARCA1 protein (NP_001269804.1); *Hs2*: *H. sapiens* SMARCA5 protein (NP_003592.3); *Sc1*: *Saccharomyces cerevisiae* Isw2p protein (NP_014948.1), *Sc2*: *Saccharomyces cerevisiae* Isw1p protein (NP_009804.1); *Dm*: *Drosophila melanogaster* (NP_523719.1); *Xl*: *Xenopus laevis* (XP_018097820.1); *At1*: *Arabidopsis thaliana* protein 11 (NP_001189826.1); *At2*: *Arabidopsis thaliana* protein 17 (NP_568365.2).

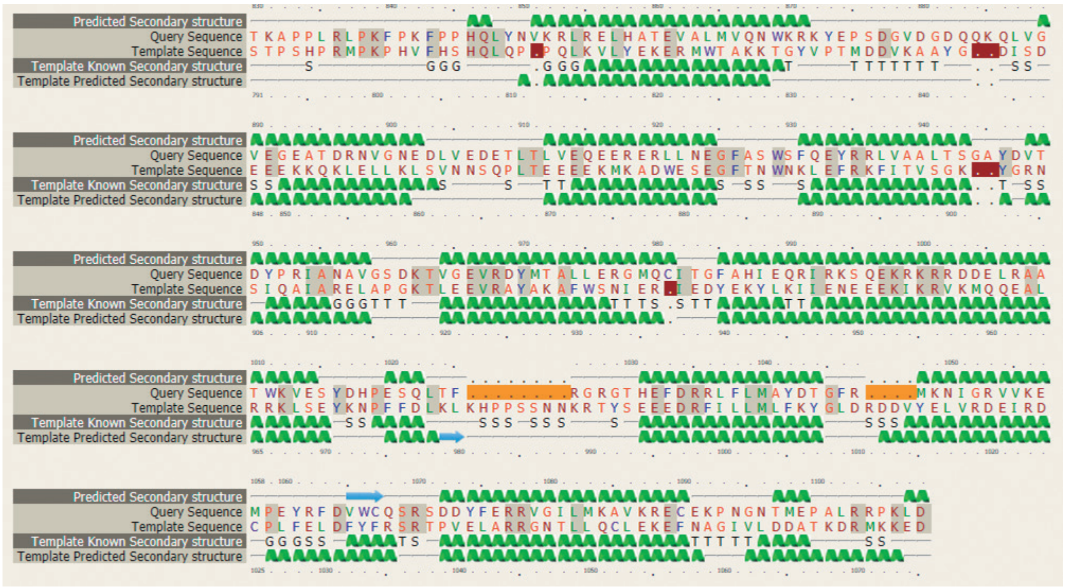

Fig. 2: secondary structure prediction of SANT domain in *Tc*ISWI. Modeling of *Tc*ISWI was performed using Phyre2. Our query had their secondary structure predicted using c2y9zA template (273 aligned residues), an ISWI protein (ISW1a) from *Saccharomyces cerevisiae*. The region comprising the amino acids 830-1108 (in the query sequence) was predicted with 100% confidence. It is possible to observe the structure similarity along the sequence, including the region corresponding to the SANT domain (926-970 residues in the query sequence) that is predicted for the template sequence.

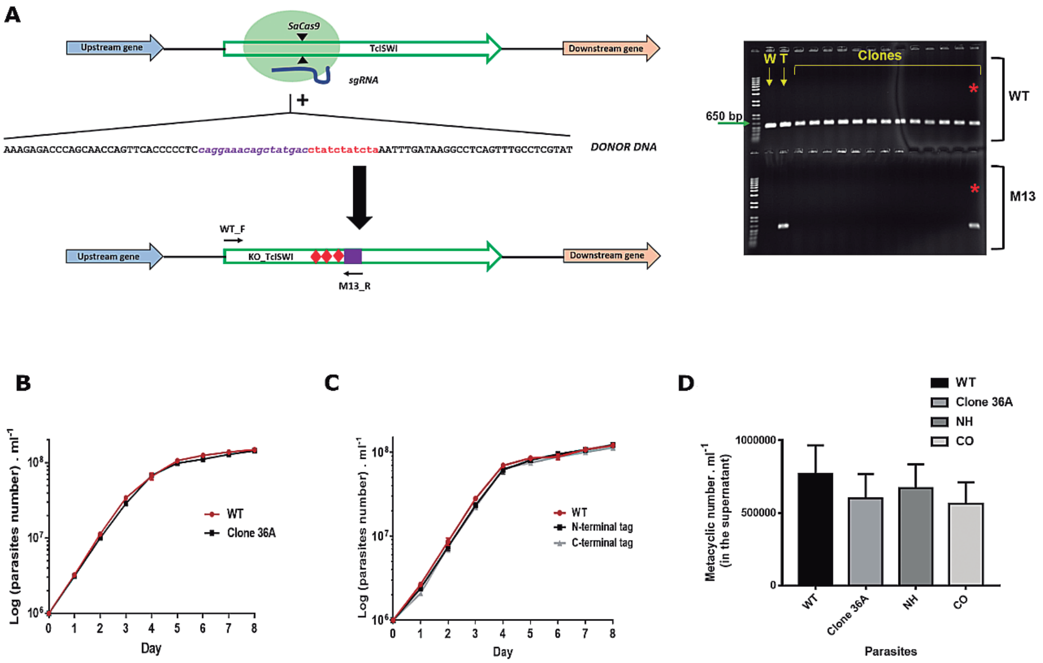

Fig. 3: phenotype evaluation in heterozygous knockout and in green fluorescent protein (GFP)-tagged *Tc*ISWI expressing parasites. (A) CRISPR-Cas9 was used to add stop codons (red diamonds and sequence) and annealing sequence for M13-R primer (purple box and sequence) to the *Tc*ISWI gene sequence, hence gene editing was assessed via polymerase chain reaction (PCR). The image in the left shows a graphic representation of the editing system, including the modifications added to the *Tc*ISWI sequence by homology-directed repair using a donor template and the position of the primers to evaluate the gene editing. The image in the right shows the PCR results. We used the WT_F and WT_R primers to amplify wild type genotype (upper region in agarose gel image) and WT_F and M13_R to amplify edited genotype (down region in agarose gel image). Primer sequences are in Supplementary data (Table I). Of all clones analysed, we obtained only one single knockout (Clone 36A) (indicated with asterisks) since both genotypes were amplified. As PCR controls, we used DNA isolated from wild type parasites (W) and parasites after transfection procedure (T). (B-C) Growth curve of the simple knockout parasites (Clone 36A) and parasites expressing GFP-tagged *Tc*ISWI protein in N-terminal and C-terminal end, respectively. Cell proliferation in transfected parasites culture was compared with that from wild type (WT) parasites. Cultures were started with a cell density of 1 x 10^6 parasites ml^{-1} and were followed by eight days. Curves represent the mean of the parasites number ml^{-1} of one experiment carried out with three technical replicates. (D) Metacyclic parasites in the supernatant of cultures after 72 h post-induction of differentiation process of single knockout and GFP-tagged *Tc*ISWI expressing parasites. Histograms show the mean of the metacyclic trypomastigotes number ml^{-1} in the supernatant of three independent experiments performed in duplicate with standard deviation indicated by error bars.

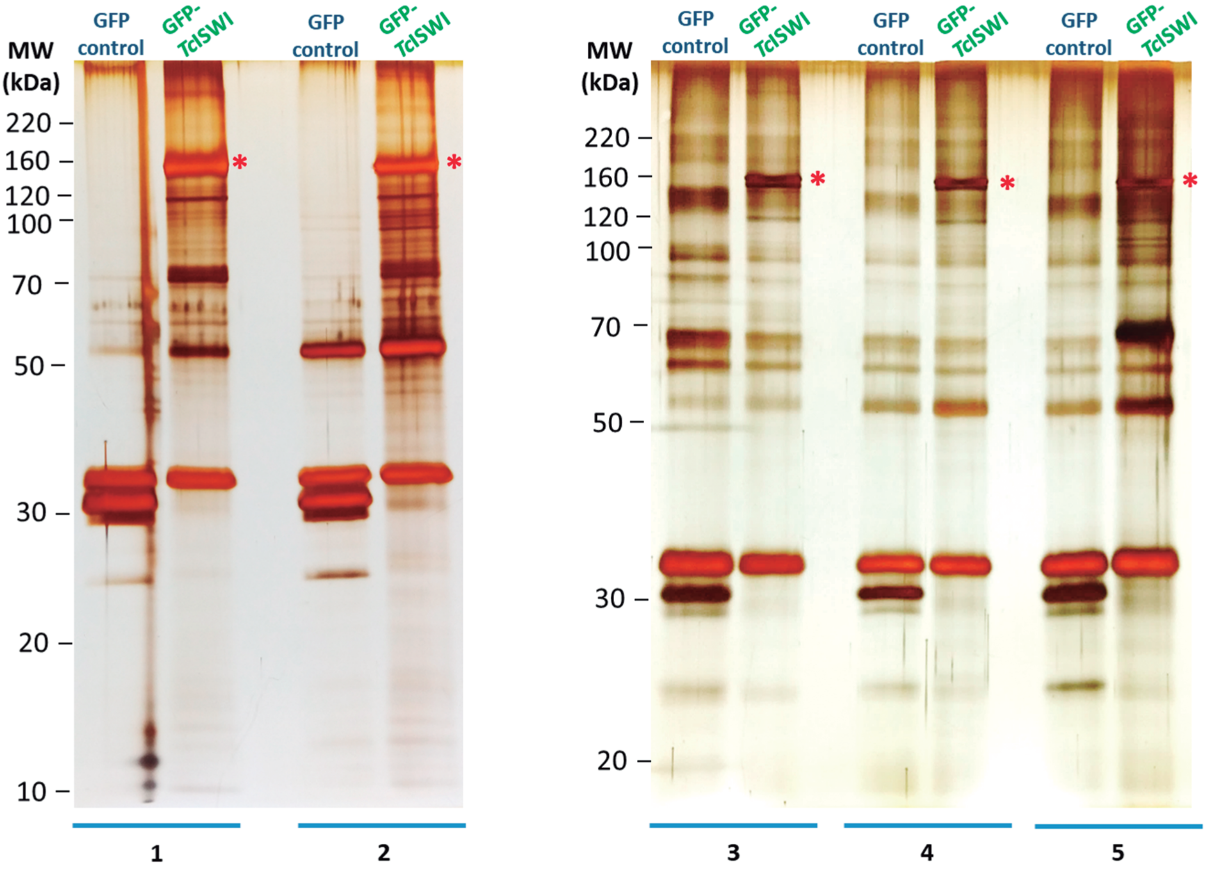

Lysis buffer components	1	2	3	4	5
**Buffer solution**	40 mM Hepes-Na pH 7.4	40 mM Tris-Cl pH 8.0	20 mM Hepes-Na pH 7.4	40 mM Tris-Cl pH 8.0	-
**Salts**	50 mM citrate<br>1 mM $MgCl_2$<br>10 µM $CaCl_2$	250 mM NaCl	250 mM citrate<br>150 mM NaCl<br>1 mM $MgCl_2$<br>10 µM $CaCl_2$	250 mM citrate<br>150 mM NaCl	500 mM ammonium acetate
**Detergent**	0,1% Triton X-100	0,1% Tween 20	1% Triton X-100	1% Triton X-100	1% Triton X-100
**Other**	10% glycerol	-	10% glycerol	-	-
**Proteases inhibitor coctel**	+	+	+	+	+

Fig. 4: optimisation of the affinity purification procedure for green fluorescent protein (GFP)-tagged *Tc*ISWI and its partners. Different lysis conditions were evaluated (lysis buffer 1 to 5) to isolate GFP-tagged *Tc*ISWI (red asterisk). Efficient isolation of GFP-tagged *Tc*ISWI was achieved using condition 1 as assessed after the resolution of the eluted proteins from capture beads by sodium dodecyl sulfate-polyacrylamide gel electrophoresis (SDS-PAGE) and silver staining. Using lysis buffer 1 was possible to observe clear differences in pattern of bands between the control sample (GFP control) and the target sample (GFP-*Tc*ISWI) and minimisation of background bands.
